# Short-chain fatty acids and type 2 diabetes: a bibliometric analysis of knowledge structure, research hotspots, and future directions

**DOI:** 10.3389/fmicb.2025.1697421

**Published:** 2025-11-12

**Authors:** Likun Zheng, Rongsheng Jiang, Jinxu Fang, Yi Tan, Yan Wang, Lian Li, Mingjun Liu

**Affiliations:** 1 College of Acupuncture and Tuina, Changchun University of Chinese Medicine, Changchun, China; 2 College of Integration of Chinese and Western Medicine, Changchun University of Chinese Medicine, Changchun, China

**Keywords:** short-chain fatty acids, type 2 diabetes, gut microbiota, metabolomics, microbial metabolism, dietary intervention, bibliometric analysis

## Abstract

**Background:**

Type 2 diabetes mellitus (T2DM) is a public health challenge that urgently needs to be addressed globally. Short-chain fatty acids (SCFAs), as metabolic products of gut microbiota, have increasingly attracted attention for their role in the pathogenesis of T2DM. This study employs bibliometric and visual analysis methods, aiming to systematically depict the knowledge structure, research hotspots, and future directions in this field.

**Method:**

We screened 965 publications on SCFAs and T2DM from the Scopus and Web of Science Core Collection databases. Visual analysis was conducted by using tools such as VOSviewer and CiteSpace.

**Result:**

Since 2016 publications in this field have increased rapidly. China leads in publication volume, while the United States plays a central role in collaboration and academic influence. Key contributors include Max Nieuwdorp and Fredrik Bäckhed. King's College London shows strong academic impact, and *Nature* is the most influential journal. Keyword analysis highlights the importance of “gut microbiota,” “metabolomics,” “microbial metabolism,” “insulin resistance,” and “dietary intervention.” These topics suggest future research will focus on signaling pathways, personalized nutrition, and microbial interventions.

**Conclusions:**

The current research focuses on a deep exploration of the role and mechanism of SCFAs in T2DM, and it aims to transition from basic research to clinical research. In terms of application, clinical workers are shifting from macroscopic dietary intervention to more precise dietary formulation strategies. The development of technologies such as metabolomics is expected to provide more powerful support for basic research and promote further progress in this field.

## 1 Introduction

T2DM is a chronic metabolic disorder that has become a significant global public health issue (Danaei et al., 2011). It is defined by hyperglycemia, insulin resistance, and low-grade inflammation, predominantly impacting the metabolism of glucose, lipids, and proteins. This chronic metabolic imbalance is believed to arise from the interplay of genetic and environmental variables, encompassing diet, lifestyle, and gut flora. According to credible sources, approximately 828 million adults worldwide were estimated to have diabetes in 2022, the vast majority being type 2 diabetes, with prevalence increasing markedly in both men and women compared with 1990 (Zhou et al., 2024). Numerous consequences, such as kidney failure, neuropathy, and cardiovascular disease, are closely linked to T2DM (Stratton, 2000; Lu et al., 2024). Not only does it severely reduce patients' quality of life and increase their economic burden, but it also imposes enormous pressure on the medical system (Cannon et al., 2018).

In the past few years, there has been a surge in the number of studies that have emphasized the importance of the intestinal microbiota and its metabolites in the development of T2DM (Canfora et al., 2019; Hosomi et al., 2022). SCFAs represent one of the principal metabolites of the gut microbiota, consisting primarily of acetate, propionate, and butyrate. They are generated under the anaerobic conditions of the intestine through the microbial fermentation of fermentable carbohydrates such as dietary fiber, resistant starch, and oligosaccharides (Cummings et al., 1987). Current studies suggest that SCFAs regulate host metabolism through multiple pathways and thereby influence the progression of T2DM (Lin et al., 2012; Yamashita et al., 2007). On one hand, SCFAs tend to activate specific G protein-coupled receptors (GPCRs) (Li et al., 2018), trigger intestinal L cells to generate GLP-1 and PYY (Tolhurst et al., 2012), increase the production of insulin, and improve insulin sensitivity (Drucker, 2018). On the other hand, they help to maintain the intestinal barrier's effectiveness (Kelly et al., 2015) and successfully lessen intestinal endotoxin leaks into the circulation, which lowers the systemic inflammatory response (Zhang and Mo, 2023). Moreover, the gut's pH can drop due to SCFAs generation, establishing an acidic milieu that is detrimental to pathogenic bacteria while promoting the proliferation of beneficial bacteria, thus aiding in the preservation of long-term metabolic equilibrium (Den Besten et al., 2013). These mechanisms collectively demonstrate the pivotal role of SCFAs in regulating T2DM (Mukhopadhya and Louis, 2025; Pham et al., 2024).

The number of studies on SCFAs and T2DM continues to increase. However, previous studies have not systematically summarized global trends, core hotspots, or evolving directions in this field. Bibliometric analysis, by evaluating publication output, collaboration networks, and keyword co-occurrence, can reveal the knowledge structure and emerging frontiers of a research domain (Jiang et al., 2023). In medical research, this approach has been extensively employed to identify prevalent research trends and offer recommendations for future paths (Ganti et al., 2025). Therefore, this study combines bibliometric and visualization strategies for performing a system evaluation of publications on SCFAs and T2DM from 1995 to 2024 to provide researchers with a macroscopic perspective and evidence to inform future investigations.

## 2 Methods

### 2.1 Sources of information and search methods

**Figure 1:**
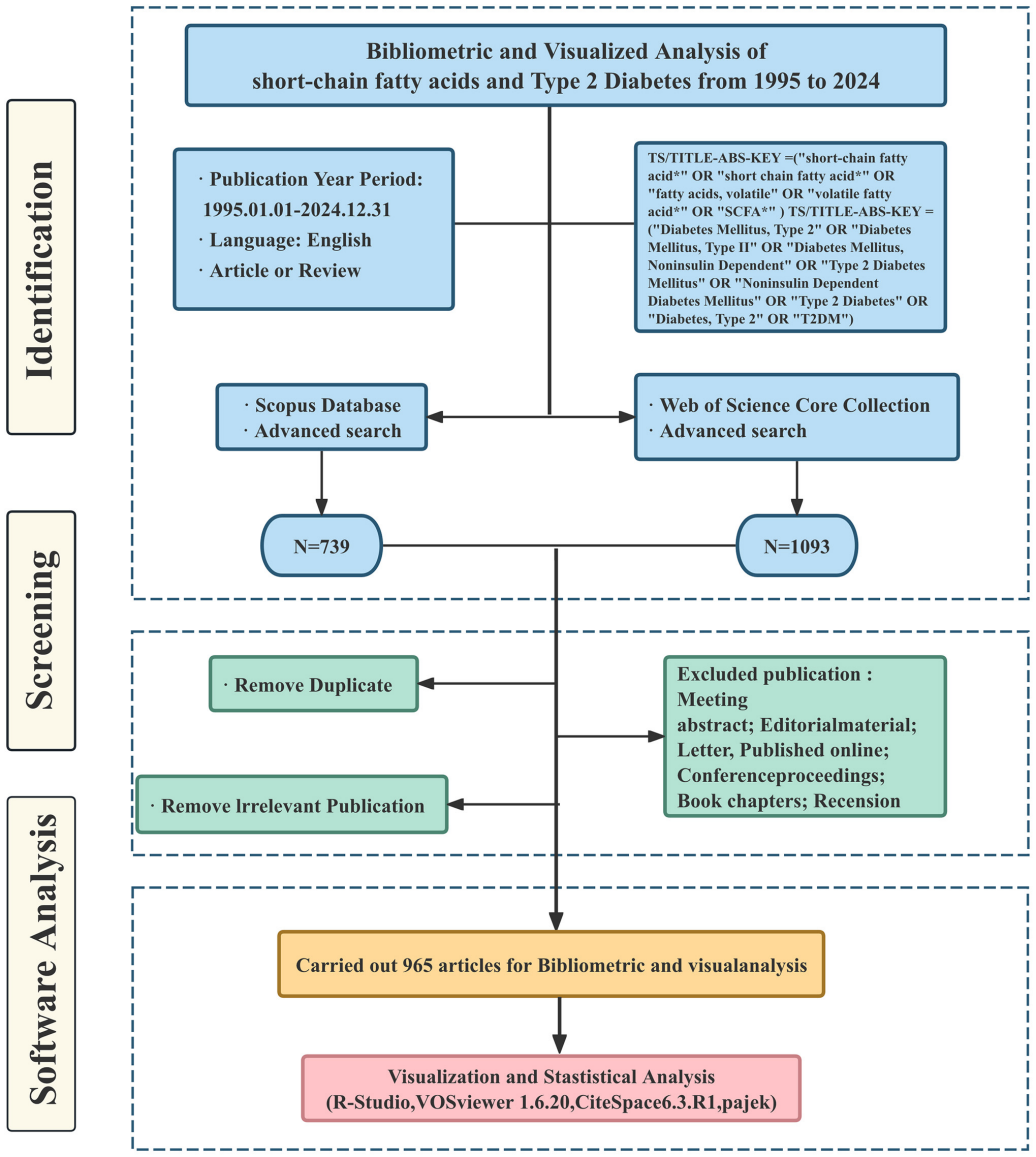
Schematic of data collection and screening.

The WoSCC and Scopus databases were the sources of information for these publications. The search was conducted on May 10, 2025. The main search terms included SCFAs and T2DM, along with related free-text terms. Table 1 describes each database's unique search approach. The search encompassed publications published from January 1, 1995, until December 31, 2024. Figure 1 illustrates the comprehensive screening process.

**Table 1:** Search strategies and results for each database.

**Databases**	**Search strategies**
Scopus	#1 TITLE-ABS-KEY = (“short-chain fatty acid^*^” OR “short chain fatty acid^*^” OR “fatty acids, volatile” OR “volatile fatty acid^*^” OR “SCFA^*^”)
	#2 TITLE-ABS-KEY = (“Diabetes Mellitus, Type 2” OR “Diabetes Mellitus, Type II” OR “Diabetes Mellitus, Non-insulin Dependent” OR “Type 2 Diabetes Mellitus” OR “Non-insulin Dependent Diabetes Mellitus” OR “Type 2 Diabetes” OR “Diabetes, Type 2” OR “T2DM”)
	#3 Document Type = Article or Review
	#4 Language = English
	#5 PY = (1995–2024)
	#6 #1 AND #2 AND #3 AND #4 AND #5
	#7 #6 NOT TITLE-ABS-KEY = (“type 1 diabet^*^” OR T1DM OR “gestational diabet^*^”)
Web of science core collection	#1 TS = (“short-chain fatty acid^*^” OR “short chain fatty acid^*^” OR “fatty acids, volatile” OR “volatile fatty acid^*^” OR “SCFA^*^”)
	#2 TS = (“Diabetes Mellitus, Type 2” OR “Diabetes Mellitus, Type II” OR “Diabetes Mellitus, Non-insulin Dependent” OR “Type 2 Diabetes Mellitus” OR “Non-insulin Dependent Diabetes Mellitus” OR “Type 2 Diabetes” OR “Diabetes, Type 2” OR “T2DM”)
	#3 Document Type = Article or Review Article
	#4 Language = English
	#5 PY = (1995–2024)
	#6 #1 AND #2 AND #3 AND #4 AND #5
	#7 #6 NOT TS = (“type 1 diabet^*^” OR T1DM OR “gestational diabet^*^”)

### 2.2 Literature screening and data extraction

Records from Scopus were exported as complete entries in CSV format, while records exported from WoSCC were obtained as “complete records and cited references” in plain text format. All the data was downloaded in a single day to maintain consistency. Two researchers (ZLK and JRS) independently screened the literature. They reviewed titles, abstracts, and references, then cross-checked all included studies.

Inclusion criteria: (1) Research type: Publicly published original research articles (Article) or systematic reviews/review articles (Review) (2) Research Object/Topic: The research must simultaneously involve the following two core contents: ① Research related to SCFAs, such as their levels, metabolism, or mechanisms of action ② Human studies, animal models, or cellular mechanism studies related to T2DM. (3) Language: English. (4) Publication period: January 1, 1995, to December 31, 2024.

Exclusion criteria for publications: (1) Publication type: Exclude conference abstracts, editorials, letters, reviews, book chapters, and other non-research or non-review literature. (2) Literature not related to the research content, for example: ① The application of SCFAs in non-biomedical fields such as ruminant nutrition, wastewater treatment, and industrial fermentation ② Type 1 diabetes, gestational diabetes, or other types of diabetes, and T2DM have not been clearly distinguished or discussed. ③ SCFAs have not been directly measured or discussed. Studies that only briefly mention “gut microbiota” or “dietary fiber” (3) cannot obtain the full text of the literature.

### 2.3 Data analysis

Bibliometric analysis employs mathematical and statistical methods. This method is considered a quantitative analysis methodology that reveals the hotspots and present condition of a study topic while forecasting its future development. This approach enables a comprehensive assessment of research trajectories across several aspects, among them research hotspots, networks of collaboration, and temporal trends (Barrington et al., 2023). R-Studio is used to plot the annual growth trend of publications (Aria and Cuccurullo, 2017), and SCImago Graphica (version 1.0.39) is used to analyze the scientific research output and cooperation pattern of countries and regions (Hassan-Montero et al., 2022). VOSviewer (version 1.6.20) is dedicated to building and measuring co-occurrence/collaboration/co-citation networks (country, institution, journal, author, keyword co-occurrence) and outputting overlay views (Van Eck and Waltman, 2010); CiteSpace (version 6.3.R1) is used for keyword burst analysis (Chen, 2004); Pajek only performs layout and beautification on the large-scale network exported by VOSviewer, without changing any weights or metrics, to enhance the readability of the image (Chen et al., 2025).

## 3 Results

### 3.1 Annual publication trends

**Figure 2:**
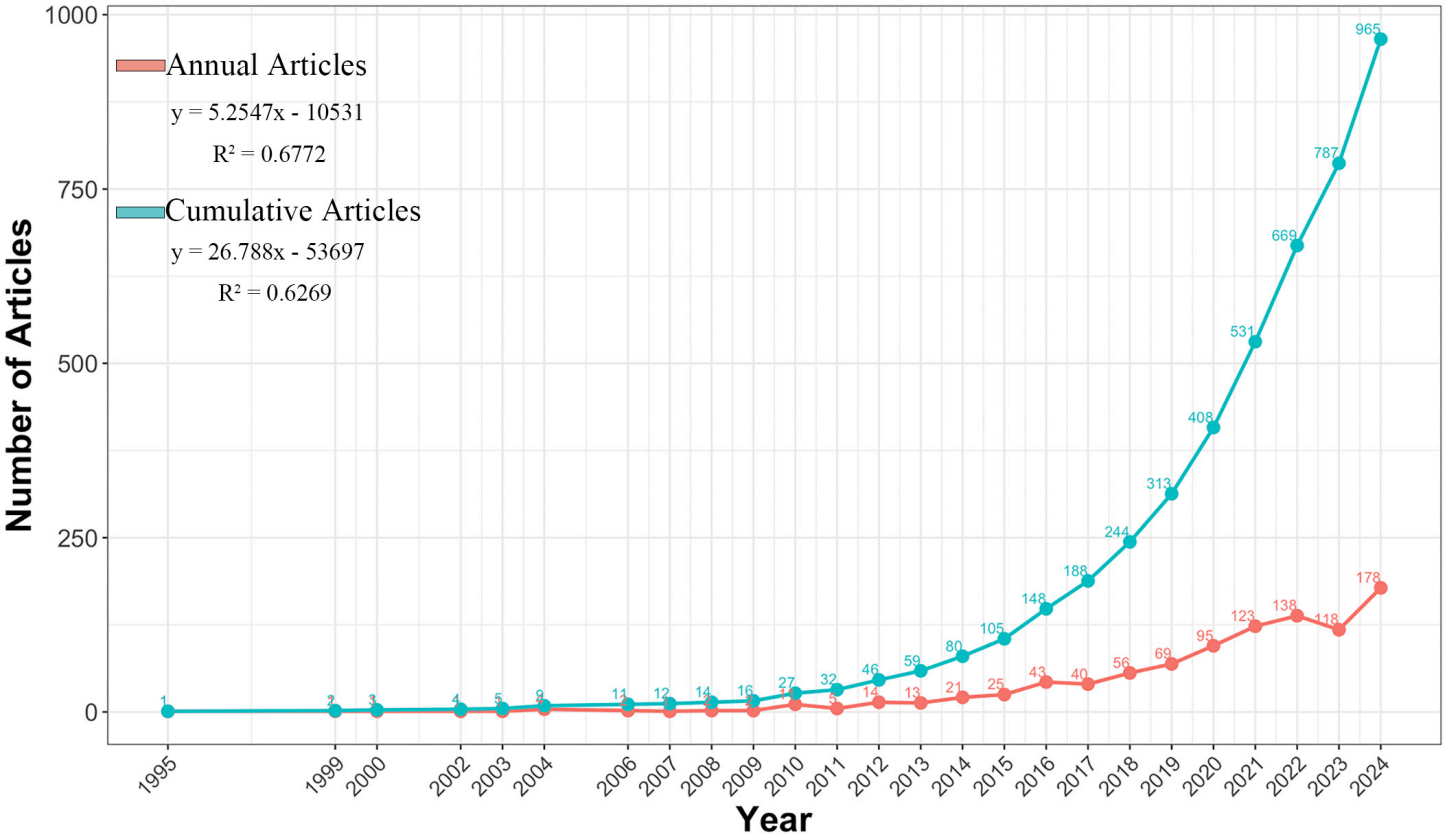
The annual and cumulative publication number from 1995 to 2024.

Figure 2 shows the changing trends of the annual and cumulative publication volumes in this field from 1995 to 2024, which can be divided into three stages: (1) The start-up period (1995–2009): The output was relatively small; (2) Initial growth period (2010–2014): Output steadily increased; (3) Rapid expansion period (2015–2024): Witness explosive growth. The two linear regression models in the figure describe the overall average situation of historical data, corresponding to “annual articles” and “cumulative articles,” respectively. Here, y represents the number of articles published annually or the cumulative total of articles up to a certain year, and x represents the year. The linear regression model for the annual publication volume is *y* = 5.2547x−10531. The coefficient of determination is *R*^2^ = 0.6772. The linear regression model of the cumulative publication volume is y = 26.788x−53697, and the coefficient of determination is *R*^2^ = 0.6269. This field saw a major rise in research over the last 10 years. The upward trend is expected to continue.

### 3.2 Country and regional analysis

Publications on T2DM and SCFAs come from 75 different countries. Table 2 lists the 10 nations in the world that produce the largest quantities. China ranked first with 407 papers (42.18%), followed by the United States with 135 papers (13.99%) and the Netherlands with 53 papers (5.49%). The United Kingdom and Australia ranked fourth and fifth, with 50 papers (5.18%) and 40 papers (4.15%), respectively. In terms of total citation counts, China leads with 20,587 citations, followed by the United States with 15,766 and the Netherlands with 12,300. Regarding the intensity of international cooperation, the United States ranks first with a total link strength of 86, followed by China with 64 and the United Kingdom with 58. Finally, in terms of citations per publication (CPP), Sweden ranks first, with the Netherlands and Germany in second and third place, respectively.

**Table 2:** Top 10 countries/regions by publication volume.

**Rank**	**Label**	**Documents *n* = 965**	**Citations**	**CPP**	**Total link strength**
1	China	407 (42.18%)	20,587	50.58	64
2	USA	135 (13.99%)	15,766	116.79	86
3	Netherlands	53 (5.49%)	12,300	232.08	55
4	UK	50 (5.18%)	8,824	176.48	58
5	Australia	40 (4.15%)	2,770	69.25	35
6	Spain	38 (3.94%)	5,830	153.42	50
7	Canada	36 (3.73%)	4,678	129.94	18
8	Italy	36 (3.73%)	3,429	95.25	29
9	Sweden	34 (3.52%)	9,288	273.18	40
10	Germany	32 (3.32%)	7,143	223.22	38

**Figure 3:**
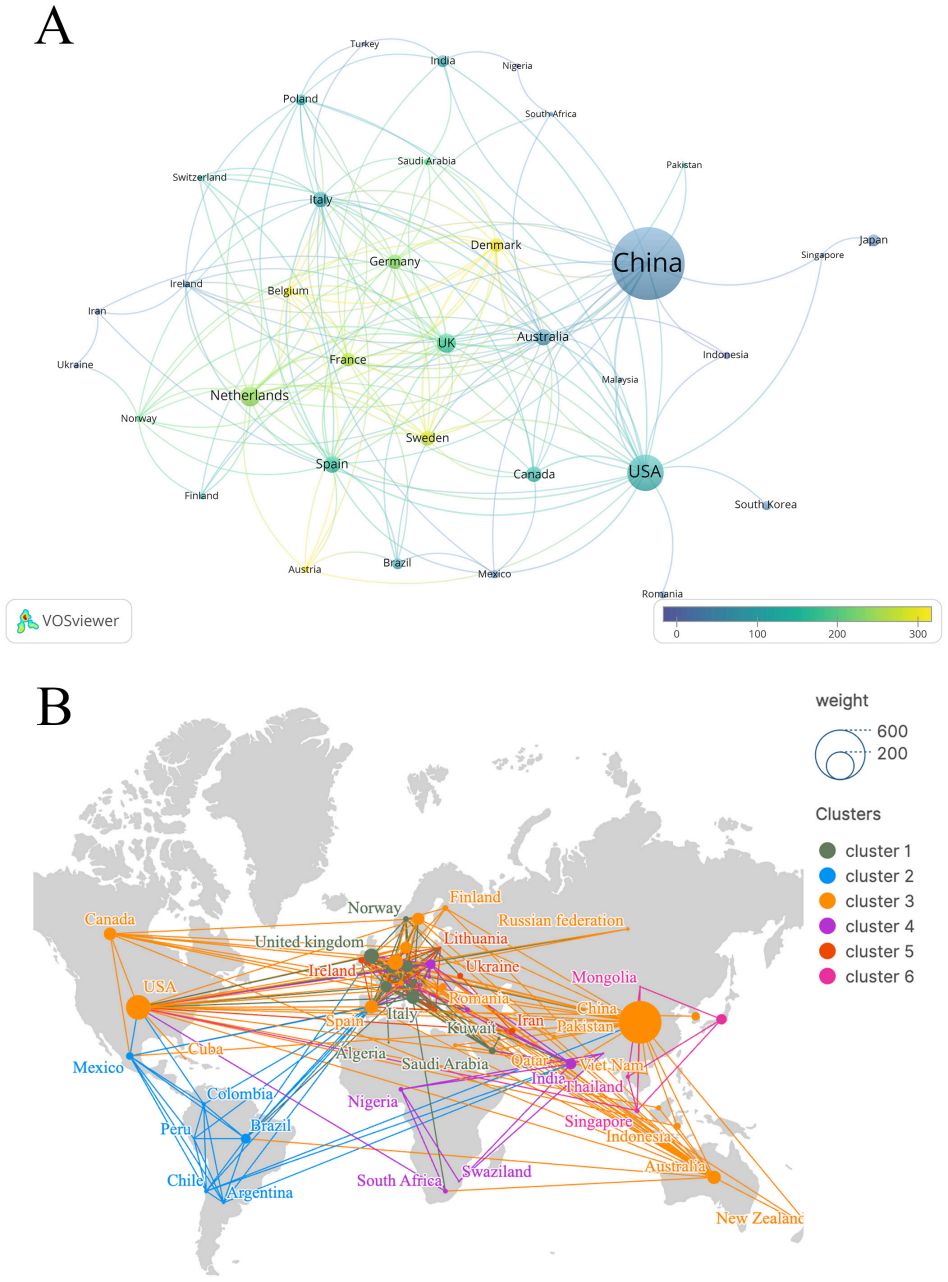
Country/region visual analysis. **(A)** Country/region co-authorship of publication and citation overlay map. **(B)** World map of countries/regions' distribution in this field.

Figure 3A shows the international collaboration network generated with VOSviewer. Each node represents a country or region, with node size weighted by publication volume. The thickness of the connecting lines indicates the strength of collaboration. The visualization uses publication volume (documents) as the weighting parameter, while the color scale reflects the average citation impact of each country. Lighter colors indicate higher average citation influence. Figure 3B further illustrates the global co-occurrence of countries and regions. The results show that this research topic is distributed worldwide, reflecting broad international attention and participation.

### 3.3 Author analysis

**Figure 4:**
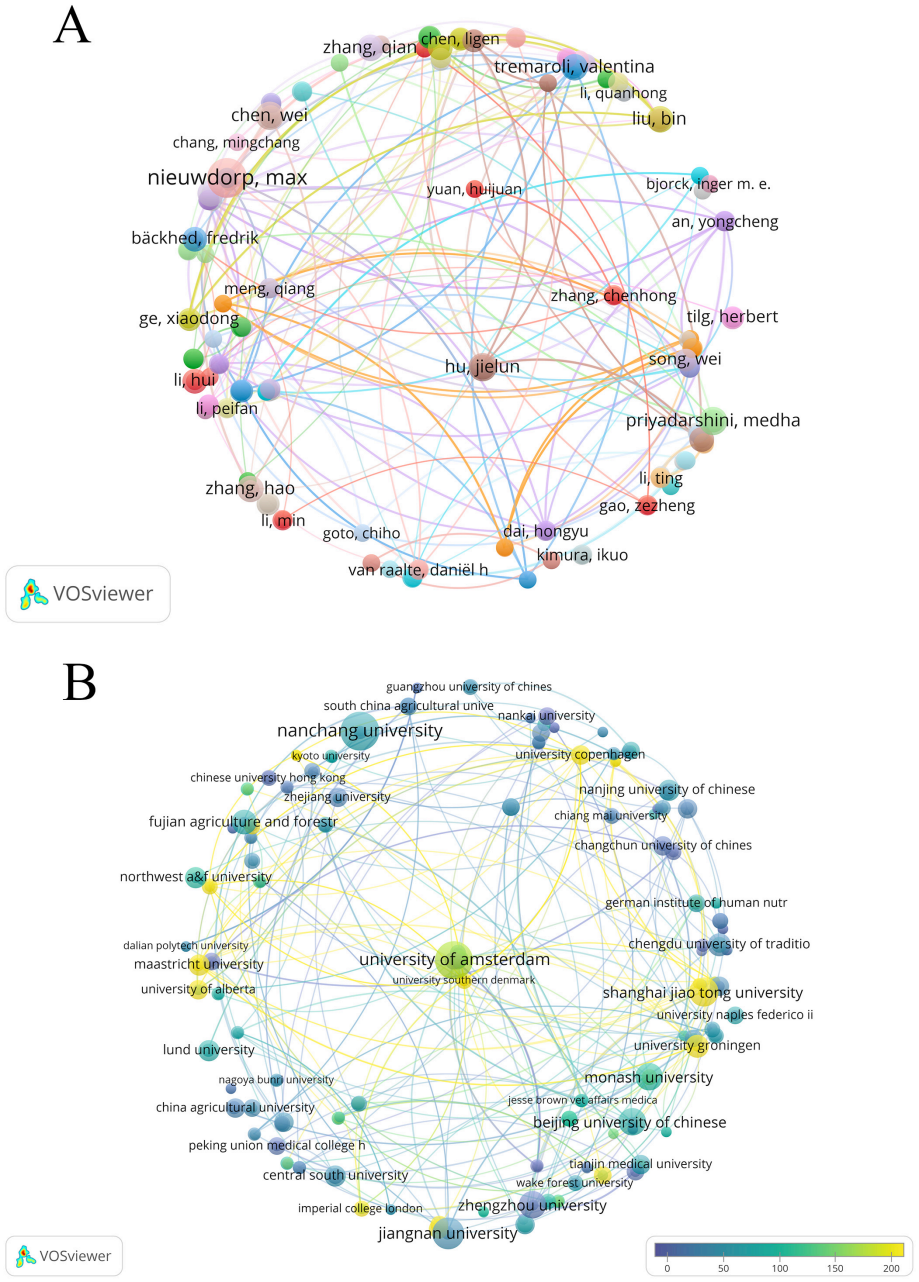
**(A)** Map visualizing author co-occurrence. **(B)** Organization of publication and citation overlay map.

Research on T2DM and SCFAs has been provided by 5,147 authors overall. The top 10 most productive authors' publication and citation statistics were gathered in Table 3, who collectively published 82 papers. As shown in Figure 4A, nodes represent authors, and the size of nodes represents the number of published articles. Dutch scholar Max Nieuwdorp ranked first with 17 publications. Chinese scholars Wei Chen and Jielun Hu, along with U.S. scholar Medha Priyadarshini, each published eight papers, tying for second place. Among the top 10 prolific authors, six were from China, which shows the remarkable activity of Chinese scholars in this field.

**Table 3:** Top 10 authors by publication volume.

**Rank**	**Author**	**Documents**	**Country/Region**	**Institutions**	**Citations**
1	Max Nieuwdorp	17	Netherlands	University of Amsterdam	2,355
2	Wei Chen	8	China	Jiangnan University	509
3	Jielun Hu	8	China	Nanchang University	817
4	Medha Priyadarshini	8	USA	University of Illinois Chicago	369
5	Bin Liu	7	China	Fujian Agriculture and Forestry University	176
6	Valentina Tremaroli	7	Sweden	University of Gothenburg	5,208
7	Xinhua Xiao	7	China	Peking Union Medical College Hospital	283
8	Hao Zhang	7	China	Jiangnan University	379
9	Qian Zhang	7	China	Peking Union Medical College Hospital	274
10	Fredrik Bäckhed	6	Sweden	University of Gothenburg	7,753

The frequency of citations is a crucial indicator for assessing an author's impact. This survey revealed 75 writers with more than 1,000 citations, with Table 4 displaying the 10 most often cited experts. Swedish scholar Fredrik Bäckhed leads with 7,753 citations, indicating significant academic influence; Valentina Tremaroli of the same institution ranked second with 5,208 citations. Austrian scholar Herbert Tilg and Dutch scholar Max Nieuwdorp ranked third and fourth with 2,448 and 2,355 times respectively. Notably, Max Nieuwdorp and Valentina Tremaroli appear on both the most-productive and most-cited lists, underscoring their significant contributions to this field.

**Table 4:** Top 10 authors by citation rate.

**Rank**	**Author**	**Citations**	**Documents**	**Country/Region**	**Institutions**
1	Fredrik Bäckhed	7,753	6	Sweden	University of Gothenburg
2	Valentina Tremaroli	5,208	7	Sweden	University of Gothenburg
3	Herbert Tilg	2,448	5	Austria	Medical University of Innsbruck
4	Max Nieuwdorp	2,355	17	Netherlands	University of Amsterdam
5	Koen Venema	2,106	5	Netherlands	Maastricht University
6	Willem Meindert de Vos	1,939	4	Netherlands	Wageningen University
7	Edward C. Deehan	1,854	3	Canada	University of Alberta
8	Kassem Makki	1,843	1	Sweden	University of Gothenburg
9	Jens W. Walter	1,843	1	Ireland	University College Cork
10	Nathalie M. Delzenne	1,816	4	Belgium	Université catholique de Louvain

### 3.4 Institutional analysis

A total of 1,303 institutions' relevant publications were retrieved in this study (Figure 4B). Each node represents an institution, with node size indicating the number of published articles and color scales reflecting the average citations per article. Lighter colors denote higher average citation impact. In terms of the number of published articles (Table 5), Nanchang University of China ranked first with 20 articles, followed closely by University of Amsterdam of the Netherlands with 19 articles. In terms of academic influence, King's College London in the UK has the highest citation count, with a total of 3,369 citations, followed by the University of Amsterdam in the Netherlands with 3,191 citations (Table 6). The University of Amsterdam not only ranks second in the number of published papers, but also performs outstandingly in citation influence, indicating that it has both output and influence in this field.

**Table 5:** Top 10 institutions by publication volume.

**Rank**	**Organization**	**Documents**	**Citations**	**Total link strength**	**Country**
1	Nanchang University	20	1,342	10	China
2	University of Amsterdam	19	3,191	28	Netherlands
3	Jiangnan University	14	585	32	China
4	Shanghai Jiao Tong University	12	2,967	23	China
5	Monash University	11	1,070	23	Australia
6	Beijing University of Chinese Medicine	11	898	12	China
7	Zhengzhou University	11	164	18	China
8	Fujian Agriculture and Forestry University	9	670	16	China
9	University of Groningen	8	2,891	23	Netherlands
10	Chengdu University of Traditional Chinese Medicine	8	271	9	China

**Table 6:** Top 10 institutions by citation rate.

**Rank**	**Organization**	**Documents**	**Citations**	**Total link strength**	**Country**
1	King's College London	4	3,369	29	UK
2	University of Amsterdam	19	3,191	28	Netherlands
3	Maastricht University	7	3,074	16	Netherlands
4	Shanghai Jiao Tong University	12	2,967	23	China
5	University of Groningen	8	2,891	23	Netherlands
6	Medical University of Innsbruck	5	2,448	13	Austria
7	Katholieke Universiteit Leuven	2	2,239	23	Belgium
8	Wageningen University & Research	6	2,036	11	Netherlands
9	King Abdulaziz university	4	2,020	22	Saudi Arabia
10	University of Copenhagen	6	1,948	26	Denmark

It is notable that the University of Amsterdam, Shanghai Jiao Tong University, and University of Groningen appear both among the top 10 institutions by publications and the top 10 institutions by citations. This shows that these institutions have outstanding overall strength in the field.

### 3.5 Journal analysis

**Figure 5:**
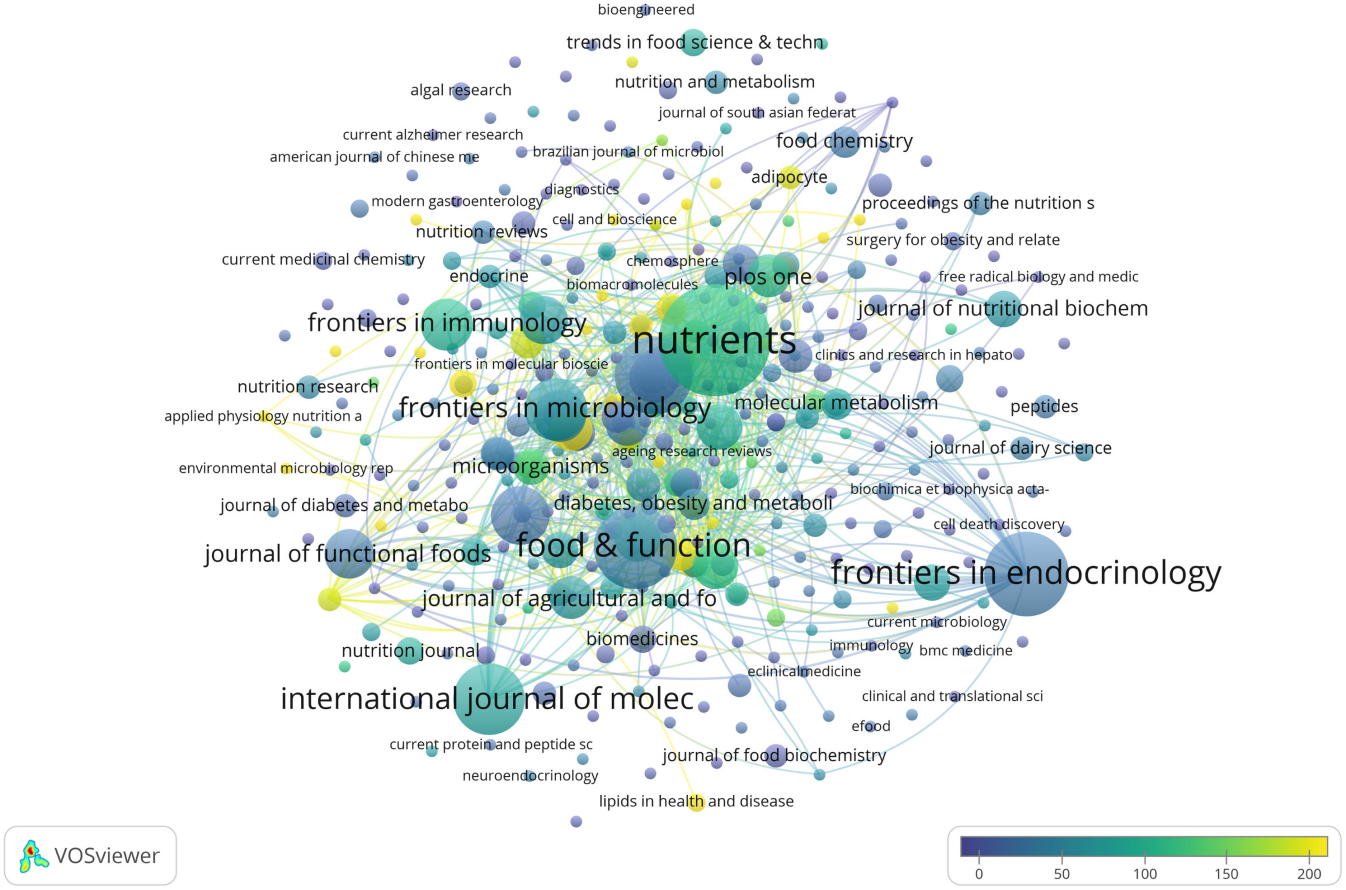
An overlay visualization of the number of journal citations.

During the past nearly 30 years, research on SCFAs and T2DM has been published across 398 journals. A network representation of these journals can be seen in Figure 5, nodes represent journals, node size indicates the number of published articles, and color scales reflect the average number of citations per article for each journal. The lighter the color, the higher the average citation influence of the research.

Table 7 enumerates the 10 journals with the highest number of publications. In terms of publication volume, the journal Nutrients ranked first with 51 papers (H-index = 75), followed by Frontiers in Endocrinology with 31 papers (H-index = 52) and Food & Function with 30 papers (H-index = 53). These three journals also ranked among the highest in total link strength, indicating their strong academic cohesion and influence within the field.

**Table 7:** Top 10 journals by publication volume.

**Rank**	**Journal**	**Documents**	**Citations**	**Total link strength**	**IF2024**	**H-Index**
1	Nutrients	51	5,617	117	5	75
2	Frontiers in Endocrinology	31	1,026	74	4.6	52
3	Food & Function	30	1,188	89	5.4	53
4	International Journal of Biological Macromolecules	27	851	24	8.5	101
5	International Journal of Molecular Sciences	23	1,794	38	4.9	114
6	Frontiers in Microbiology	18	1,142	45	4.5	88
7	Frontiers in Nutrition	16	423	36	5.1	–
8	Frontiers in Immunology	13	1,278	12	5.9	84
9	Food Research International	13	576	30	8	134
10	Journal of Functional Foods	12	388	37	4	63

In terms of citation impact (Table 8), Nature published only three papers but received 7,003 citations, with 2,334 references annually for each article, ranking first. Nutrients ranked second, with 51 papers accumulating 5,617 citations (an average of 110 citations per paper). Gut ranked third, with four papers receiving 2,605 citations (an average of 651 citations per paper). Science contributed only two papers but achieved 2,430 citations (an average of 1,215 citations per paper).

**Table 8:** Top 10 journals by citation rate.

**Rank**	**Journal**	**Citations**	**Documents**	**Total link strength**	**IF2024**	**H-Index**
1	Nature	7,003	3	52	48.5	1,096
2	Nutrients	5,617	51	117	5	75
3	Gut	2,605	4	12	25.8	262
4	Science	2,430	2	51	45.8	1,058
5	Cell Host & Microbe	2,049	3	11	18.7	147
6	British Journal of Nutrition	1,971	9	51	3	166
7	International Journal of Molecular Sciences	1,794	23	38	4.9	114
8	Nature Reviews Endocrinology	1,661	5	20	40	116
9	Nature Medicine	1,573	2	40	50	497
10	Circulation Research	1,286	1	2	16.2	306

### 3.6 Keyword analysis

**Figure 6:**
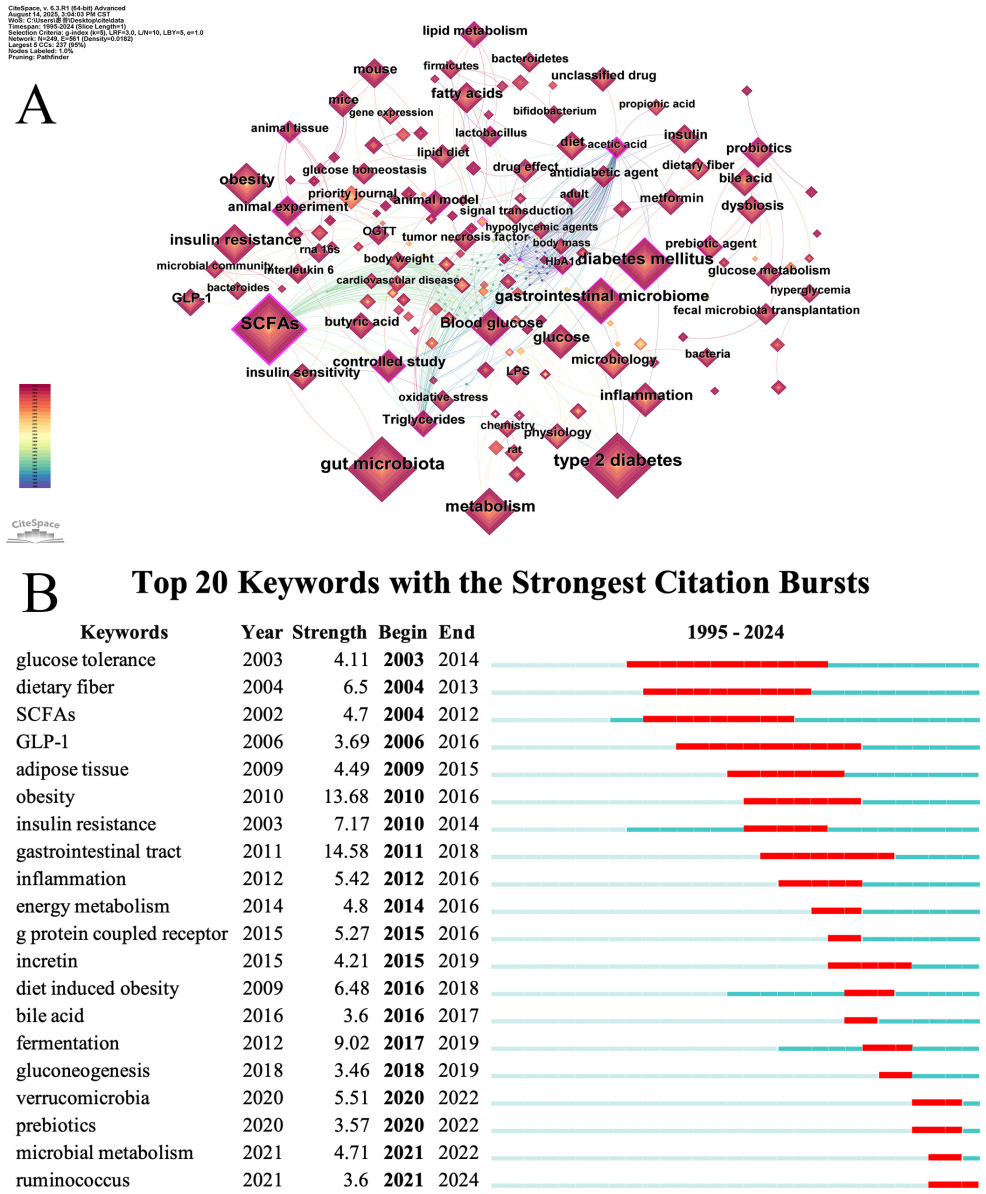
**(A)** Map visualizing keyword co-occurrence. **(B)** Top 20 view of keywords bursts analysis.

Key words reflect the main idea and core content of publications. Through co-occurrence and cluster analysis of key words, the distribution of research topics, field hotspots, and frontier directions can be revealed (Zhong et al., 2022). Figure 6A shows nodes representing keywords, with node size indicating the frequency of keyword occurrence. Table 9 presents the top 20 keywords of Co-occurrence Frequency, among which the most common keyword is “SCFAs” (*n* = 726), “type 2 diabetes” (*n* = 709), and “gut microbiota” (*n* = 663), which together represent the central themes of this field. Other high-frequency keywords included “glucose” (*n* = 386), “metabolism” (*n* = 352), “obesity” (*n* = 350), “gut microbiome” (*n* = 317), “insulin resistance” (*n* = 281), “diet” (*n* = 269), and “inflammation” (*n* = 230).

**Table 9:** Top 20 keywords of co-occurrence frequency.

**Rank**	**Keyword**	**Occurrences**	**Total link strength**
1	SCFAs	726	18,440
2	type 2 diabetes	709	18,349
3	gut microbiota	663	16,179
4	glucose	386	11,728
5	metabolism	352	10,481
6	obesity	350	9,193
7	gut microbiome	317	10,015
8	insulin resistance	281	8,458
9	diet	269	6,811
10	inflammation	230	6,438
11	glucose blood level	195	7,567
12	probiotics	179	5,652
14	microbiology	151	5,084
15	mouse	150	5,410
16	dysbiosis	147	5,141
17	GLP-1	146	4,662
18	animal experiment	130	5,547
19	lipid metabolism	127	5,187
20	insulin sensitivity	126	4,286

**Figure 7:**
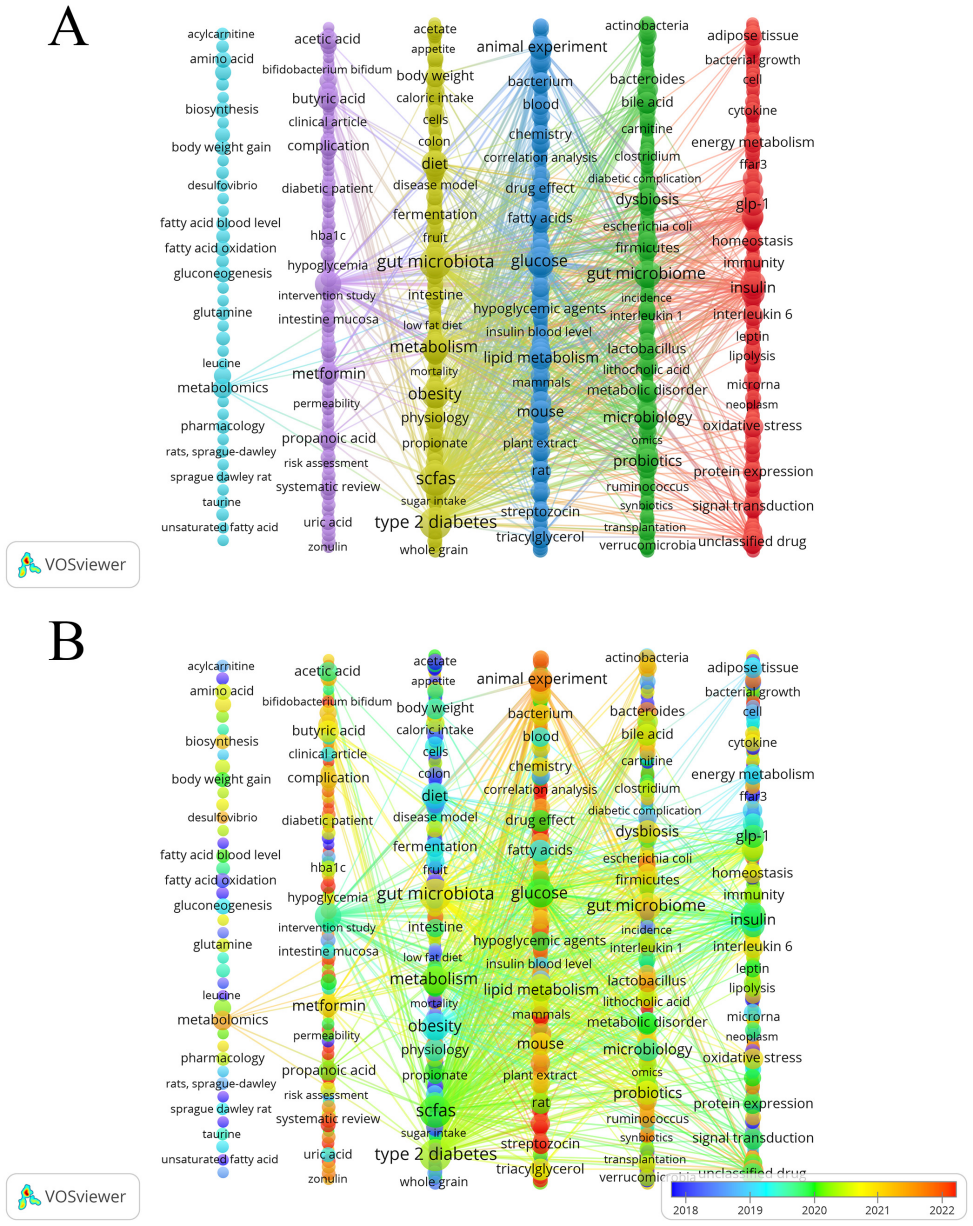
**(A)** Keywords clustering visualization. **(B)** Keywords intensity visualization timing overlay.

We generated a keyword co-occurrence map using VOSviewer and refined the layout in Pajek to improve visual clarity. This process identified six clusters. As shown in Figure 7A, each node represents a keyword. The node size indicates the frequency of the keyword, and different colors mark different thematic clusters. The themes were identified through an integrated analysis of the most frequent and most central keywords within each cluster. The yellow cluster highlighted the core of the research field, focusing on dietary interventions involving SCFAs in relation to T2DM.Keywords include “diet,” “whole grain,” “low fat diet,” “fruit,” “fermentation,” and “sugar intake.” The green cluster focuses on gut microbiota and microbiology, examining their roles in SCFAs production and T2DM. Keywords include “bacteroides,” “firmicutes,” “lactobacillus,” “ruminococcus,” “probiotics” and “synbiotics.” The Red Cluster focuses on SCFAs regulating T2DM-related signaling pathways. Keywords include “ffar3,” “glp-1,” “insulin,” “leptin,” “interleukin 6,” and “signal transduction.” The blue cluster (middle right) focused on animal experiments investigating the function of SCFAs in glucose metabolism. Keywords include “animal experiment,” “mouse,” “rat,” “streptozotocin,” and “correlation analysis.” The Purple Research Group has examined the impact of acetate, propionate, and butyrate, which are specific SCFAs, on T2DM. Keywords include “acetic acid,” “butyric acid,” “propanoic acid,” “intestinal mucosa,” “permeability,” and “zonulin.” The light-blue cluster (far left) concentrated on metabolomics approaches, underscoring their importance in elucidating the relationship between SCFAs and T2DM. Core keywords include “metabolomics” and common small-molecule analytes in metabolomics, such as “amino acid,” “acylcarnitine,” “leucine,” and “taurine.”

By overlaying time on the keyword clustering map, we analyzed changes in research directions within the field. Figure 7B shows the average publication year of keywords. The color of each node represents the average year when the keyword became a research hotspot. Blue indicates earlier occurrence, while yellow indicates more recent occurrence. In the early stage (blue nodes), keywords such as “acylcarnitine,” “unsaturated fatty acid,” “acetate,” “ffar3,” “colon,” and “fatty acid oxidation.” During the early-to-middle transitional stage (blue-green nodes), keywords such as “diet,” “obesity,” “cell,” “taurine,” “Sprague Dawley rat,” and “whole grain.” In the middle stage (green nodes), keywords such as “acetic acid,” “glp-1,” “insulin,” “leptin,” “lipolysis,” “metabolic disorder,” “microbiology,” “scfas,” “type 2 diabetes,” “glucose,” and “protein expression.” In the later stage (yellow–red nodes), keywords such as “bifidobacterium bifidum,” “systematic review,” “animal experiment,” “streptozocin,” “synbiotics,” and “correlation analysis.”

Burst keyword analysis can reveal the emergence, development, and decline of research hotspots and help researchers identify emerging fields, frontier directions, and future trends (Li, 2017). As shown in Figure 6B, the red bar chart to the right of each keyword indicates the duration of the outbreak for that keyword. The figure displays 20 burst keywords in studies of SCFAs and T2DM. The keyword “gastrointestinal tract” exhibited the strongest burst intensity (Strength = 14.58), followed by “obesity” (Strength = 13.68) and “fermentation” (Strength = 9.02). “Glucose tolerance” is the keyword with the highest continuous attention.

### 3.7 Citation analysis

**Figure 8:**
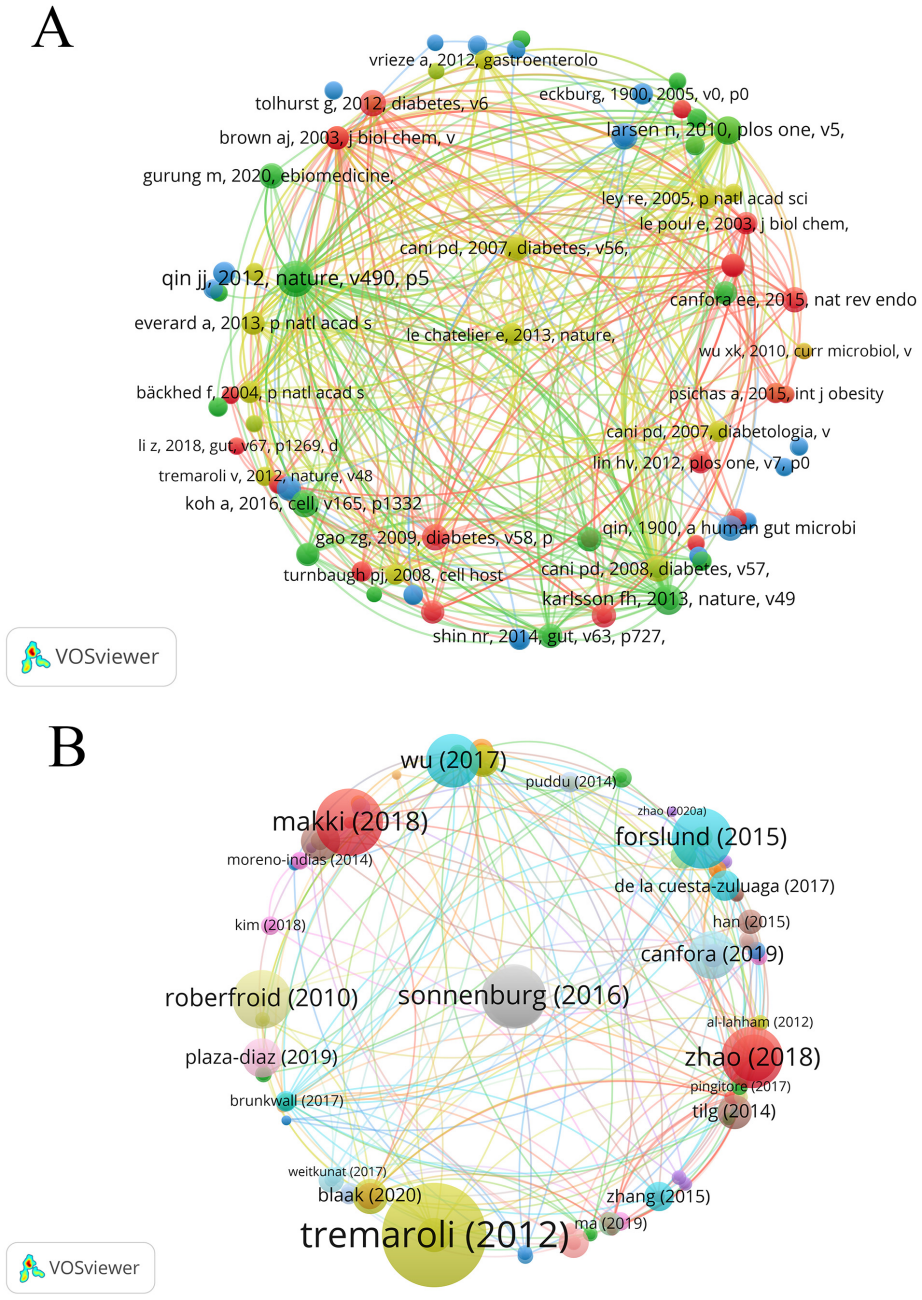
**(A)** Co-citation analysis cited reference. **(B)** Map visualizing cited publications.

A total of 20,131 references were cited among the 965 included publications, with 44 of those citations occurring more than 20 times. Table 10 enumerates the 10 most co-cited references in the domain of SCFAs and T2DM, comprising nine research articles and one review, with co-citation counts ranging from 37 to 90. As shown in Figure 8A, each node represents a publication. The size of a node reflects the number of citations. The larger the node, the higher the number of citations and the greater the influence. The lines connecting nodes indicate the co-citation relationship. The most frequently co-cited reference was a scholarly paper in Nature by Qin et al. (2012) (impact factor 48.5), which was co-cited 90 times.

**Table 10:** Top 10 references by co-cited.

**Rank**	**First author**	**Journal**	**Title**	**Publication type**	**Citations**	**IF2024**
1	Junjie Qin	Nature	A metagenome-wide association study of gut microbiota in type 2 diabetes	Article	90	48.5
2	Fredrik H. Karlsson	Nature	Gut metagenome in European women with normal, impaired and diabetic glucose control	Article	56	48.5
3	Nadja Larsen	Plos one	Gut microbiota in human adults with type 2 diabetes differs from non-diabetic adults	Article	45	2.6
4	Liping Zhao	Science	Gut bacteria selectively promoted by dietary fibers alleviate type 2 diabetes	Article	44	45.8
5	Ara Koh	Cell	From Dietary Fiber to Host Physiology: Short-Chain Fatty Acids as Key Bacterial Metabolites	Review	41	42.5
6	Gwen Tolhurst	Diabetes	Short-chain fatty acids stimulate glucagon-like peptide-1 secretion via the G-protein-coupled receptor FFAR2	Article	40	7.5
7	Zhanguo Gao	Diabetes	Butyrate improves insulin sensitivity and increases energy expenditure in mice	Article	39	7.5
8	Peter J. Turnbaugh	Nature	An obesity-associated gut microbiome with increased capacity for energy harvest	Article	39	48.5
9	Patrice D. Cani	Diabetes	Changes in gut microbiota control metabolic endotoxemia-induced inflammation in high-fat diet-induced obesity and diabetes in mice	Article	38	7.5
10	Patrice D. Cani	Diabetes	Metabolic endotoxemia initiates obesity and insulin resistance	Article	37	7.5

Across all publications, 84 articles received more than 200 citations. Table 11 lists the top ten most cited works, including three articles and seven reviews. Figure 8B shows the node size, which reflects the citation frequency of the publication. The larger the node, the higher the number of citations and the greater the impact, the largest node corresponds to the review by Tremaroli published in Nature (Tremaroli and Bäckhed, 2012), which has been cited 3,689 times, ranking first in Table 10 with an impact factor of 48.5. The second and third positions are occupied by the reviews authored by Makki et al. (2018) and Sonnenburg and Bäckhed (2016), with 1,843 and 1,749 citations, respectively.

**Table 11:** Top 10 publications by citations.

**Rank**	**First author**	**Journal**	**Title**	**Publication type**	**Citations**	**IF_2024_**
1	Valentina Tremaroli	Nature	Functional interactions between the gut microbiota and host metabolism	Review	3,689	48.5
2	Kassem Makki	Cell Host and Microbe	The impact of dietary fiber on gut microbiota in host health and disease	Review	1,843	18.7
3	Justin L. Sonnenburg	Nature	Diet–microbiota interactions as moderators of human metabolism	Review	1,749	48.5
4	Liping Zhao	Science	Gut bacteria selectively promoted by dietary fibers alleviate type 2 diabetes	Article	1,633	49.5
5	Kristofer Forslund	Nature	Disentangling type 2 diabetes and metformin treatment signatures in the human gut microbiota	Article	1,565	48.5
6	Fabien Magne	Nutrients	TheFirmicutes/Bacteroidetes ratio, a relevant marker of gut dysbiosis in obese patients?	Review	1,556	5
7	Marcel Roberfroid	British Journal of Nutrition	Prebiotic effects: metabolic and health benefits	Review	1,514	3
8	Willem M. de Vos	Gut	Gut microbiome and health: mechanistic insights	Review	1,502	25.8
9	Hao Wu	Nature Medicine	Metformin alters the gut microbiome of individuals with treatment-naive type 2 diabetes, contributing to the therapeutic effects of the drug	Article	1,296	50
10	Wai Hong Wilson Tang	Circulation Research	Gut microbiota in cardiovascular health and disease	Review	1,286	16.2

## 4 Discussion

### 4.1 General information

This study uses bibliometric methods to analyze research related to SCFAs and T2DM, and it can be observed that researchers' attention to this field has increased year by year in recent years. In the initial stage, the research mainly focused on the substrate metabolism related to SCFAs in T2DM, such as resistant starch and high fiber diet (Alles et al., 1999; Wolever et al., 2002). During the early stage, research gradually turned to how dietary interventions affect the production of SCFAs and to exploring their mechanisms in the development of T2DM (Tolhurst et al., 2012; Cornall et al., 2013). After 2015, the development of technologies such as metabolomics accelerated publication growth (Clish, 2015), accompanied by a surge of animal experiments and clinical trials. These studies explored the preventive and therapeutic effects of SCFAs or their precursors, such as dietary fiber and prebiotics, on T2DM, with growing attention to individual variability (Vliex et al., 2024; Zhang et al., 2020).

China has the largest number of publications, while the United States shows the highest total link strength. Both countries have made important contributions to this research field. It is worth noting that, in terms of average citations per paper, which reflect the average impact of research, European countries such as Sweden and the Netherlands perform better. Several factors may contribute to this. On the one hand, these countries have infrastructure such as large cohorts and microbiome sample banks, which supports high quality empirical research. For example, the Dutch Microbiome Project is a large-scale study on the function of gut microbiota (Gacesa et al., 2022). These achievements are the result of long-term accumulation. This also indicates that research in this field started earlier in these countries, and their publications have had more time to accumulate citations. On the other hand, since English is the main language in these countries and their publications are often in open access or top journals, their work is more easily noticed and cited by the international academic community.

Dutch scholar Max Nieuwdorp and Swedish scholar Fredrik Bäckhed were identified as the most productive and the most frequently cited authors, respectively. Nieuwdorp has focused on the relationship between gut microbiota and various diseases, emphasizing the regulatory role of SCFAs in human health (Baars et al., 2024; Kullberg et al., 2025). Medha Priyadarshini and Jielun Hu concentrated on SCFAs receptors (FFAR2/3, GPR109A) and their roles in glucose metabolism, intestinal barrier function, and inflammation (Priyadarshini et al., 2016; Nie et al., 2020). Xinhua Xiao and Qian Zhang investigated the mechanisms of drug-mediated modulation of gut microbiota (Guo et al., 2025; Ding et al., 2022). Wei Chen and Hao Zhang explored probiotics and functional foods (Zhu et al., 2025), while Bin Liu focused on dietary polysaccharides and hepatic pathways in improving insulin resistance and lipid metabolism (Lee et al., 2024).

We found that the highly cited scholars mainly concentrate their research interests in the following areas. Fredrik Bäckhed, Valentina Tremaroli, Herbert Tilg, and Max Nieuwdorp focus on animal experiments, human intervention studies, and review analyses, seeking to elucidate the processes by which gut microbiota influence host metabolism (Tremaroli and Bäckhed, 2012; De Vos et al., 2022; Wu et al., 2017). Edward C. Deehan, Kassem Makki, Jens Walter, Nathalie M. Delzenne, and Koen Venema primarily investigate how dietary fibers and prebiotics enhance SCFAs production, with particular attention to their applications in nutritional interventions and metabolic regulation (Den Besten et al., 2013; Makki et al., 2018; Seethaler et al., 2022; Yang et al., 2013). Willem M. de Vos emphasizes the identification of key functional bacteria and their translational applications, which have advanced the practical implementation of microbiome research findings (Zhang et al., 2024).

From the perspective of institutional distribution, seven of the top 10 institutions by publication volume are from China, highlighting the contribution of Chinese institutions in this field. Among the top 10 institutions by citation count, four are from the Netherlands, reflecting the strong academic influence of Dutch institutions in this area. Among the top 10 journals in terms of publication volume, five focus on food science and nutrition, two on molecular biology, and the remaining three belong to the fields of endocrinology, microbiology, and immunology, respectively. These journals have a strong disciplinary correlation with each other, reflecting the interdisciplinary characteristics of this research topic. And although Nature and Science have published relatively few papers in this field, their high citation rates highlight their leading academic influence.

Co-citation analysis of references facilitates the rapid accumulation of domain knowledge and helps identify research dynamics. In this study, the top 10 most frequently cited 10 references were further analyzed and categorized into four groups based on their research content. (1) Population-based observational studies: Larsen et al. (2010) utilized 16S pyrosequencing and qPCR in Danish adult males, revealing a substantial integration of the relative number of particular bacterial phyla and plasma glucose concentrations. Qin et al. (2012) used shotgun metagenomic sequencing in Chinese adults and found gut microbiota dysbiosis in T2DM patients, distinguished by a significant decrease in bacteria that produce butyrate. Karlsson et al. (2013) investigated European women and, through metagenomic clustering (MGCs), reported heightened prevalence of Lactobacillus and diminished prevalence of Clostridium in people with T2DM. (2) Clinical dietary intervention studies: Zhao et al. intervened in Chinese T2DM patients through a high-fiber diet, combined with fecal microbiota transplantation experiments, revealed an increase in 15 SCFA-producing bacterial strains. These changes were closely associated with reductions in HbA1c, elevations in GLP-1 and PYY, and improvements in glycemic control (Zhao et al., 2018). (3) Mechanistic studies: Tolhurst et al. (2012) conducted experimental studies incorporating wild-type mice and FFAR2/FFAR3 knockout mice. They demonstrated that acetate, propionate, and butyrate promote GLP-1 secretion via FFAR2/3, thereby influencing glucose metabolism. Gao et al. (2009) supplemented butyrate in mice subjected to a high-fat diet and discovered that it enhanced energy expenditure and improved insulin sensitivity. Cani et al. (2007, 2008), through mouse experiments, revealed that bacterial lipopolysaccharide (LPS) levels were closely associated with inflammation, metabolic disturbances, and endotoxemia. Turnbaugh et al. (2006) compared obese and lean mice, demonstrating that concentrations of acetate and butyrate were markedly increased in the cecum of fat mice. (4) Review studies: Koh et al. (2016) offered an in-depth investigation of the origins and targets of SCFAs, emphasizing that butyrate and propionate can suppress histone deacetylases (HDACs) to modulate gene transcription. SCFAs can also directly activate GPCRs, thereby modulating multiple signaling pathways and host physiological functions.

We further analyzed the top 10 most cited publications and found that their research themes were primarily centered on diet mediating the microbiota, generating SCFAs, and regulating the chain of metabolic processes and associated mechanisms. Diet composition and microbial metabolism: Tremaroli and Bäckhed (2012); Makki et al. (2018); Sonnenburg and Bäckhed (2016); Zhao et al. (2018) focused on the influence of dietary structure on gut microbiota and metabolic outcomes, providing theoretical support for the dietary fiber-driven modulation of SCFAs production Mechanistic insights: De Vos et al. (2022) and Tang et al. (2017) systematically described the mechanisms of SCFAs in T2DM, indicating that SCFAs activate GPR41/43 to promote the production of PYY and GLP-1, consequently affecting hunger, energy metabolism, and insulin release. Prebiotics and gut environment: Roberfroid et al. (2010) emphasized the health effects of prebiotics, noting that fermentable fibers can alter the gut environment (e.g., reduce fecal pH) and modulate the SCFA profile. Drugs and microbiota remodeling: Forslund et al. (2015) and Wu et al. (2017) reported that metformin and other medications reshape the gut microbiota, with part of their metabolic effects mediated through alterations in community structure. Microbiota composition and disease associations: Magne et al. (2020) analyzed the correlation between gut microbial makeup and disease progression, emphasizing how varying community patterns may influence the onset of metabolic problems.

The research in the above-mentioned hotspot literature mainly focuses on dietary interventions, such as increasing dietary fiber intake or supplementing with probiotics, to regulate the composition and function of the gut microbiota and promote the production of key metabolites such as SCFAs. Acting as core signaling molecules, SCFAs activate GPCRs and inhibit HDACs, thereby regulating the host's hormone secretion, glucose and lipid metabolism, and inflammatory responses. This series of studies, from population observation, clinical intervention to molecular mechanism, jointly confirmed the key role of SCFAs in regulating T2DM.

### 4.2 Research hotspots

Keyword analysis can reveal the research hotspots of the discipline and reflect the knowledge structure. We summarize the research hotspots in the field by interpreting these six clusters, which are summarized into three parts, they are, respectively the generation of SCFAs, the research on the mechanism of SCFAs mediating T2DM, and the application of omics technology.

#### 4.2.1 Generation of SCFAs

The core of the yellow cluster, “dietary intervention,” and the core of the green cluster, “gut microbiota,” jointly constitute the regulatory axis of SCFAs generation. Research indicates that high-fiber dietary patterns, exemplified by the Mediterranean diet, can significantly increase SCFA levels, enhance intestinal barrier integrity, and diminish plasma concentrations of lipopolysaccharide-binding protein, thereby contributing to the regulation of T2DM onset and progression (Seethaler et al., 2022; Salamone et al., 2021). These studies point the way to the modulation of T2DM through dietary promotion of SCFAs production. At the same time, the production of SCFAs is closely related to the composition of gut microbiota, and enhancing the beneficial bacteria that produce SCFAs in the gut helps maintain host metabolic homeostasis (Antony et al., 2023). Supplementing with probiotics or prebiotics can increase the abundance of beneficial bacteria such as Lactobacillus and Bifidobacterium in the gut, thereby raising SCFAs levels (Li et al., 2021). In recent studies, “prebiotics” can serve as a key dietary factor. By promoting the growth of probiotics and optimizing the intestinal environment, they can increase the production of SCFAs such as acetic acid and butyric acid, playing a crucial role in improving insulin secretion and regulating appetite (Kume et al., 2018; Baba et al., 2025) The core of this process is “microbial metabolism,” which refers to the gut microbiota producing a variety of bioactive products, including SCFAs, by breaking down substrates such as dietary fiber and prebiotics (Baba et al., 2025; Myhrstad et al., 2020). Emerging research hotspots further focus on specific functional bacteria, such as Akkermansia muciniphila within the Verrucomicrobia phylum, which can produce acetate and propionate to enhance the gut barrier (De Vos, 2017; He et al., 2023). And the genus Ruminococcus, which is positively correlated with dietary fiber intake, has main metabolic products of butyric acid and propionic acid, which are crucial for maintaining energy metabolism (Yang et al., 2013; Van-Wehle and Vital, 2024). The production of SCFAs depends on the supply of dietary substrates and the metabolic activity of the gut microbiota. In terms of diet, particularly the intake of high fiber and prebiotics, it can influence SCFAs production by shaping the gut ecosystem.

#### 4.2.2 Research on the mechanism of SCFAs mediating T2DM

The red cluster focuses on SCFAs that regulate T2DM related signaling pathways. SCFAs can act as compounds to activate various GPCRs, including GPR41/FFAR3 and GPR43/FFAR2 (Brown et al., 2003). These receptors are widely expressed in enteroendocrine cells, pancreatic β-cells, adipose tissue, liver, and immune cells. Upon activation, they stimulate the secretion of gastrointestinal hormones, including GLP-1 and PYY, thereby increasing insulin release, inhibiting glucagon secretion, and improving glucose homeostasis (Kimura et al., 2014; González Hernández et al., 2019). Meanwhile, the purple cluster focuses on mechanistic studies of specific SCFAs, such as acetate, propionate, and butyrate. Acetate has been shown to activate the AMPK pathway, thereby promoting energy metabolism and lipid oxidation, and improving energy balance (Frost et al., 2014). Propionic acid can act as a substrate for gluconeogenesis to regulate glucose homeostasis (Todesco et al., 1991) and participate in lipid metabolism by inhibiting HMG-CoA reductase (Lin et al., 1995). Butyrate is essential for the protection of the intestinal barrier, contributing to the integrity of the intestinal epithelium and the tightness of its connections, which reduces endotoxin translocation into the bloodstream, decreases inflammation and improves insulin sensitivity (Xu et al., 2018). In addition, butyrate promotes peripherally induced Treg cell differentiation, enhances immune tolerance, and attenuates pancreatic inflammation (Hao et al., 2021). These findings emphasize the multiple roles of different types of SCFAs in energy metabolism, glucose and lipid homeostasis, intestinal barrier, and immune regulation, and provide a theoretical basis and research direction for intervening in T2DM by targeting SCFAs mechanisms. Meanwhile, the effectiveness of these mechanisms is mainly verified through animal experiments, as shown by blue clustering. A meta-analysis of SCFAs intervention in a diabetic mouse model showed that butyrate could reduce triglyceride (TG), total cholesterol (TC), and fasting blood glucose (FBG) levels in T2DM mice (Zheng et al., 2024). Overall, the related studies mainly explored the deep mechanism through animal models, and found that SCFAs can effectively regulate the onset and progression of T2DM through the physiological regulatory network of multi-level and multi-targets. At the signal transduction level, SCFAs regulates the release of enteroendocrine hormones (such as GLP-1 and PYY), insulin secretion and inflammatory response by activating GPCRs. At the metabolic level, they are involved in energy homeostasis and lipid metabolism, promote glucose utilization and improve insulin sensitivity. At the same time, SCFAs can maintain the integrity of the intestinal barrier, reduce endotoxin translocation, and inhibit chronic low-grade inflammation; its immunoregulatory effects can also promote the differentiation of anti-inflammatory cells and restore metabolic homeostasis.

#### 4.2.3 Omics technologies and applications of SCFAs in T2DM

The light blue cluster reveals the central role of metabolomics as a cutting-edge technology in advancing research in this field. Analytical techniques represented by gas chromatography–mass spectrometry (GC-MS) and liquid chromatography–mass spectrometry (LC-MS) provide essential technical support for the precise quantification of SCFAs and other metabolites, as well as for elucidating the complex interactions between the gut microbiota and the host (Saha et al., 2021; Llauradó et al., 2025). Untargeted metabolomics is also widely applied to explore the roles of microbial metabolites in metabolic diseases. For example, some researchers have revealed the mechanism by which fecal microbiota transplantation improves T2DM mouse models through an integrated study combining untargeted metabolomics and 16S rRNA analysis (Yang et al., 2023). With technological advancements, research hotspots are gradually shifting from the macroscopic “phylum” level toward analyses at the functional genus and species levels. Integrated analyses combining metabolomics, metagenomics, and transcriptomics have enabled researchers to focus on specific beneficial microbial communities.

### 4.3 Clinical translation and future trend analysis

In recent years, multiple studies have shown from different levels that interventions surrounding SCFAs production may provide new pathways for the management of T2DM. A clinical trial found that the combination of probiotics and metformin can reshape the fecal microbiome composition of patients with T2DM, especially by increasing the abundance of various SCFAs-producing bacteria. Meanwhile, their combination significantly enhances the hypoglycemic effect and increases insulin secretion, improving pancreatic function (Chen et al., 2023). A randomized crossover trial showed that short-term supplementation with multivitamins and minerals, at doses near recommended levels, also altered the gut microbiota composition and metabolite profile in healthy adults, characterized by increased levels of SCFAs such as propionate and butyrate. It is worth noting that this effect is not determined solely by the supplement but is influenced by the individual's existing dietary structure (Mckirdy et al., 2025). In terms of dietary intervention, the high-fiber diet proposed by the researchers can significantly improve the SCFAs level in feces and simultaneously improve clinical outcomes such as HbA1c, fasting blood sugar and insulin resistance; at the same time, the study also identified a group of core strains that produce acetic acid and butyric acid. Based on this, the researcher proposed the concept of “ecological therapy”: by restoring or promoting the abundance of SCFAs producing bacteria and rebalancing the intestinal microecology, the metabolic health of the host can be improved. The research also pointed out that personalized nutrition strategies can be utilized to specifically cultivate highly active SCFAs producers into “ecosystem service providers (ESP),” with the aim of managing T2DM more effectively (Zhao et al., 2018). In addition to nutritional and microbiota intervention, delivery technology is also expanding the clinical application possibilities of SCFAs. Researchers have developed the oral multifunctional carrier system “nano-in-microparticles,” which uses supercritical emulsion extraction to co-encapsulate butyric acid and propionic acid for targeted release in the colon. The findings of this study represent an innovative approach, providing a new perspective for the clinical use of SCFAs to mediate T2DM (Carvalho et al., 2025). In population studies, heterogeneity should also be considered. A randomized, double-blind, placebo-controlled, crossover clinical trial showed that short-term high-dose inulin increased SCFAs production in both young and older adults, with a more pronounced rise in butyrate. However, the increase in older adults was relatively limited (Kirschner et al., 2025). Consistent with this, the analysis of nine types of SCFAs producing bacteria genera found that their abundance significantly decreased with the aging process of the human body, and there was no significant difference between men and women (Wu et al., 2024). In addition, there are systematic differences in the composition of basic microbiota and dietary habits among different regions and populations (such as the East and the West, tropical/temperate zones, and urban and rural areas), which will significantly affect the generation potential and intervention response of SCFAs. Therefore, an effective SCFAs intervention strategy in one population may not achieve the same effect in another (Sheng et al., 2024).

Taken together, SCFAs related interventions have certain advantages in clinical applications, and there are multiple pathways to enhance the production of SCFAs *in vivo*, with the sources relying mainly on dietary fiber, prebiotics, or probiotics, with a high degree of safety and acceptability. However, clinical translation still faces many challenges, including marked interindividual differences across populations, issues of tolerance and absorption with direct SCFAs supplementation, and the lack of evidence from large-scale, long-term randomized controlled trials. In the future, with the advancement of multi-omics technologies and personalized medicine, SCFAs-related interventions are expected to find broader application in precision nutrition and combined strategies. This may drive a shift from general dietary guidelines to individualized nutrition management, offering new pathways for T2DM management and public health improvement.

### 4.4 Limitations

(1) This study was analyzed based on data from two databases, WoSCC and Scopus, so other database-related research may be missed. Future research should be included in databases such as PubMed and Embase to minimize biases caused by database selection and increase coverage. (2) Both WOSCC and Scopus primarily index high-impact, English-language international journals, so non-English research may be excluded. In future studies, we recommend developing bibliometric tools with multilingual analysis capabilities to address these shortcomings. (3) This research analysis mainly focuses on articles and reviews, and excludes other types of publications such as conference papers, book chapters, etc., which may ignore the potential important research results. Future research should integrate these diverse literature types to build a more complete and dynamic view of domain development. (4) The citation rate of articles is usually influenced by multiple factors such as self-citations. Future research should conduct longitudinal tracking of relevant citations to assess their effectiveness. (5) This study has a certain time lag in the literature; the latest research may not have been cited or included. In the future, the most recent literature can be incorporated to update the development status of the field, thereby providing a more comprehensive depiction of its evolutionary panorama. (6) By leveraging the unique advantages of different tools (CiteSpace, VOSviewer), we obtain a more comprehensive picture of the research field. However, a methodological limitation is that we did not systematically cross-validate multiple tools with different core algorithms, so the specific results may exhibit slight variations. Although we observed a high consistency in the major research themes at the macro level across analyses using different tools, future research should more systematically compare the effects of different algorithms on bibliometric results to enhance the robustness of the conclusions. (7) This study is based on published scientific literature, but it is undeniable that we did not consider publication bias. The proportion of positive findings in the literature is too high. This bias should be reduced in future research.

## 5 Conclusion

This study systematically analyzed the development trends, current hotspots, and future directions of research on SCFAs related to T2DM. The results help researchers understand the knowledge structure in this field and provide a reference for future studies.

In the future, for researchers, combining modern technologies such as metabolomics and metagenomics with multidisciplinary approaches from nutrition, microbiology, and endocrinology can be used to more deeply elucidate the specific mechanisms by which SCFAs improve T2DM. For clinical practice, optimizing SCFAs-based interventions and developing individualized treatment strategies that consider patients' ethnicity, age, and gender differences is expected to bring better clinical outcomes.

## Data Availability

The original contributions presented in the study are included in the article/supplementary material, further inquiries can be directed to the corresponding author.
